# Esmolol improves sepsis outcomes through cardiovascular and immune modulation

**DOI:** 10.3389/fphar.2025.1498227

**Published:** 2025-05-12

**Authors:** Da Jing, Li Xiong, Rong Zhang, Hong Fang, Lin Chen

**Affiliations:** Department of Pulmonary and Critical Care Medicine, Sichuan Provincial People’s Hospital, School of Medicine, University of Electronic Science and Technology of China, Chengdu, China

**Keywords:** sepsis, Esmolol, β1-adrenergic blocker, sympathetic nervous system, immune modulation, T-cell regulation

## Abstract

**Background:**

Sepsis poses significant mortality risks. Esmolol, a β1-adrenergic blocker, may improve outcomes through cardiovascular and immune modulation. This study aims to evaluate the effects of Esmolol on survival rates, inflammatory markers, and immune function in sepsis patients.

**Methods:**

In this retrospective observational study, data from 268 sepsis patients were reviewed, and 125 met the inclusion criteria. These patients were divided into Esmolol and control groups. Data were collected from electronic health records, including survival rates, inflammatory markers (IL-6, PCT), and immune function markers (CD4^+^ and CD8^+^ T-cell counts). Statistical analyses included multivariate regression, Kaplan-Meier survival analysis, and Generalized Estimating Equations.

**Results:**

The Esmolol group demonstrated significantly higher survival rates at both 14 days (80% vs. 41.67%, p < 0.01) and 28 days (75.38% vs. 30.00%, p < 0.01) compared to the control group. The median ICU stay was longer in the Esmolol group (12 days vs. 10 days, P = 0.045). Significant reductions in heart rate (P = 0.002), NE levels (P = 0.036), and inflammatory markers were observed in the Esmolol group. Additionally, Esmolol treatment resulted in bidirectional regulation of T-cell counts, increasing CD4^+^ and CD8^+^ T-cell counts in patients with higher baseline immune function and decreasing these counts in patients with lower baseline levels (P < 0.01).

**Conclusion:**

Esmolol improves survival rates and clinical outcomes in sepsis patients, particularly those with higher baseline immune function. The benefits are attributed to early and prolonged administration of Esmolol, highlighting its potential as a valuable addition to sepsis treatment protocols. Future multicenter trials are needed to confirm these findings and refine the use of β1AR in sepsis management.

**Clinical Trial Registration:** clinicaltrials.gov, identifier NCT06390748.

## 1 Introduction

Sepsis is a life-threatening condition characterized by a dysregulated immune response to infection, leading to systemic inflammation, organ dysfunction, and high mortality, particularly among elderly and immunocompromised patients. Despite advancements in critical care, sepsis remains a major challenge in intensive care unit (ICU). Its pathophysiology involves a complex interplay of pro-inflammatory and anti-inflammatory responses, often culminating in immune paralysis and increased susceptibility to secondary infections (Stolk et al., 2016; Jensen et al., 2018).

Esmolol, a short-acting selective β1-adrenergic receptor (β1AR) blocker, is widely used in acute cardiovascular management for controlling heart rate and blood pressure. Clinically, it reduces myocardial ischemia without inducing significant bradycardia or hypotension (Ollila et al., 2018), mitigates hypertension and tachycardia in hyperadrenergic states (Pollan and Tadjziechy, 1989), and improves postoperative cardiac function by lowering the inotropic score and reducing the risk of low-cardiac-output syndrome (Zangrillo et al., 2021). Its cardiovascular benefits extend to sepsis management, where it has been shown to enhance stroke volume index and reduce mortality without compromising cardiac output (Vásquez-Tirado et al., 2024; Hasegawa et al., 2021).

The functions of β1AR and β2AR are central to both cardiovascular and immune regulation. β1AR primarily regulates cardiac chronotropy and inotropy, enhancing cardiac output under stress (Rohrer et al., 1999; Bernstein, 2002; Wei and Smrcka, 2022), while β2AR modulates vascular tone, cardiac remodeling, and fibroblast proliferation (Rohrer et al., 1999; Bernstein, 2002; Chesley et al., 2000; Tanner et al., 2020). Beyond cardiovascular regulation, β2AR also plays a crucial role in immune modulation by influencing cytokine production and immune cell recruitment (Tanner et al., 2021; Kolmus et al., 2015).

Esmolol selectively blocks β1AR, reducing heart rate and myocardial contractility while sparing β2AR activity. This selectivity preserves vasodilation and bronchodilation, which is particularly advantageous in patients with asthma or peripheral vascular disease (Muller et al., 2017; Nasrollahi-Shirazi et al., 2016). Additionally, esmolol does not interfere with β2-mediated epinephrine effects, maintaining critical stress responses (Muller et al., 2017). Morelli et al. (2013) further highlight its expanding role beyond cardiovascular applications (Morelli et al., 2013).

The sympathetic nervous system (SNS) exerts significant influence over immune function, particularly through norepinephrine (NE) signaling via β2AR on T and B cells. SNS activation and NE release can deplete T cells via β1AR, suggesting that esmolol may mitigate immune suppression in sepsis by modulating NE levels and T-cell function (Scanzano et al., 2015). NE plays a dual role in immune regulation—while essential for hemodynamic stability, chronic elevation can impair immune responses by depleting T cells and increasing inflammatory cytokine production (Pedicino and Volpe, 2024). NE modulation has been shown to alter cytokine profiles, reducing TNF-α and increasing IL-10 in septic patients (Sharma and Farrar, 2020), but excessive suppression of immune activity can increase susceptibility to secondary infections, particularly in elderly patients.

Beyond its cardiovascular effects, esmolol has demonstrated potential in sepsis management by attenuating sepsis-induced immunosuppression. It has been observed to restore CD4 T-cell function and normalize regulatory T lymphocyte proportions, highlighting its immunoregulatory properties (Durand et al., 2022). This study aims to evaluate esmolol’s impact on sepsis by assessing its effects on NE levels, immune function, and inflammatory markers. We hypothesize that esmolol not only stabilizes cardiovascular function but also enhances immune resilience, reducing immune paralysis and improving clinical outcomes.

## 2 Materials and methods

### 2.1 Study design and ethical approvals

This study was conducted in accordance with the Declaration of Helsinki and approved by the Ethics Committee of Sichuan Provincial People’s Hospital (Approval No. 211). This retrospective observational study analyzed patients treated at the Department of Pulmonary and Critical Care Medicine, Sichuan Provincial People’s Hospital, with clinical data extracted from the Electronic Health Record (EHR) system.

We reviewed data from 268 sepsis patients who met the Sepsis-3 diagnostic criteria and received standard fluid resuscitation for septic shock. Exclusion criteria included patients who died within the first 3 days of ICU admission, those with severe cardiac failure (NYHA Class III or higher), long-term β-blocker use, recent ECMO or CRRT, and viral sepsis (including COVID-19) to maintain a homogeneous study population.

### 2.2 Data collection and study subgroups

Clinical data, including demographics, clinical details, Esmolol dosages, hemodynamic parameters, and daily vital signs such as heart rate, blood pressure, and NE levels, were sourced from the EHR. Data on changes in APACHE II and SOFA scores, immune function, and inflammatory markers (CD4^+^ and CD8^+^ T-cell counts, PCT, IL-6, TNF-α, IFN-γ) were also documented. The measurement of these immune markers was part of routine ICU practice in our center for assessing immune status and guiding treatment decisions. Patients were included consecutively from January 2021 to December 2023, ensuring an unbiased selection of cases within the study period. The study period was chosen to reflect a representative sample of sepsis patients managed under consistent institutional protocols. Given the retrospective nature of this study, all data were collected from existing medical records without additional interventions. Two researchers independently validated the data for reliability and consistency.

Participants were divided into two groups based on their treatment records: those who received continuous intravenous Esmolol at doses greater than 80 mg/h for more than 3 days (E group) and a control group of patients who did not receive Esmolol, matched for age, gender, and APACHE II scores. Esmolol was administered to patients with hyperadrenergic states characterized by systemic inflammatory response syndrome (SIRS) and persistent tachycardia (heart rate > 120 bpm) despite adequate volume resuscitation and vasopressor support. Only a minority of sepsis patients met the criteria for Esmolol administration.

Esmolol treatment was titrated based on heart rate response, with a target range of 80–100 bpm. The infusion rate was adjusted every 30–60 min, and the maximum dose was limited to 240 mg/h. If significant hypotension (MAP < 65 mmHg) or bradycardia (HR < 60 bpm) occurred despite vasopressor support, the infusion was reduced or discontinued. The treatment duration typically ranged from 3 to 7 days, depending on the patient’s hemodynamic stability.

The study used a stratified comparative approach to assess Esmolol’s effects on NE levels and CD4^+^ counts in sepsis patients, analyzing the data retrospectively to determine the impact of Esmolol treatment on these parameters.

### 2.3 Statistical analysis

Statistical analyses were conducted using SPSS software (version 25.0, IBM Corp., Armonk, NY, United States). Descriptive statistics were reported as mean ± standard deviation or median with 95% confidence intervals, depending on the data distribution. For comparisons between two groups, independent samples t-tests or Mann-Whitney U tests were used. For comparisons involving multiple groups, ANOVA was employed, including Bonferroni correction to manage Type I error risks.

Survival analysis was conducted using Kaplan-Meier curves, with Log-rank tests employed for group comparisons. Binary logistic regression was used to identify independent predictors of survival, with odds ratios (OR) and 95% confidence. Intervals (CI) reported. Multicollinearity was assessed using variance inflation factors (VIF), and model goodness-of-fit was evaluated using the Hosmer-Lemeshow test.

To assess predictive accuracy, Receiver Operating Characteristic (ROC) curve analysis was conducted, with the Area Under the Curve (AUC) used to quantify discrimination ability. The Esmolol variable was coded as a binary predictor (1 = Esmolol use, 0 = no use), and predicted probabilities from logistic regression were used to generate ROC curves. Additional ROC analyses were performed for APACHE II scores, NE-end levels, and a combined Esmolol + APACHE II model to evaluate the added predictive value of combining Esmolol use with clinical severity scoring. The optimal threshold was determined using the Youden index, with corresponding sensitivity and specifity values reported.

For repeated measures of immune-related indicators, such as cytokine levels (IL-6, TNF-α, IFN-γ) and T cell counts (CD4^+^, CD8^+^), Generalized Estimating Equations (GEE) models were used. GEE was selected due to its ability to account for correlated data within subjects over time, handle missing data more effectively, and avoid strict normality assumptions. All statistical tests were two-tailed, and a P-value <0.05 was considered statistically significant.

## 3 Results

### 3.1 Basic characteristics of the study population

**FIGURE 1:**
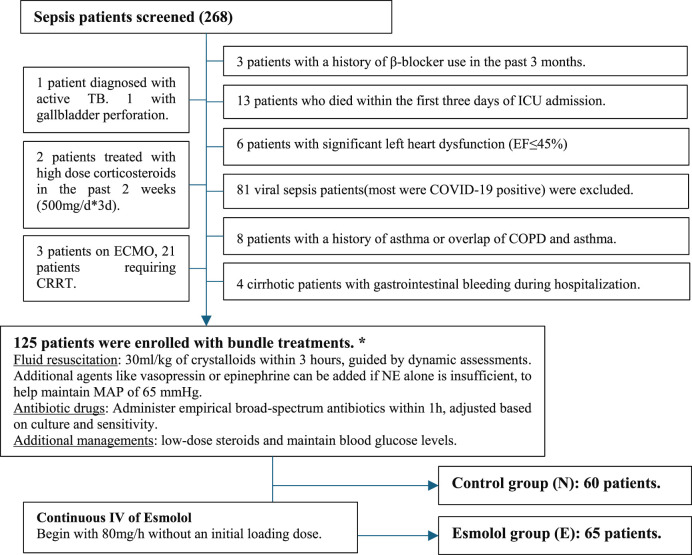
Flowchart of patient enrollment and treatment protocol in sepsis management study. Notes: The flowchart illustrates the process of patient enrollment and exclusion in the sepsis management study. Out of 268 initial sepsis patients, strict exclusion criteria were applied, resulting in 125 patients being enrolled for Boule treatments. These patients were divided into N group and E group. The Esmolol group received a continuous IV infusion starting at 80 mg/h, with gradual increases up to 240 mg/h based on blood pressure tolerance. The flowchart also details the administration of fluid resuscitation, antibiotic drugs, and additional management strategies as part of the treatment protocol. * Based on Sepsis-3.0 and the International Sepsis and Infectious Shock Management Guidelines 2021 (Surviving Sepsis Campaign Guidelines 2021). # If the patient cannot tolerate higher infusion rates due to blood pressure issues, maintain the infusion at the initial rate of 80 mg/h.

Initially, 268 sepsis patients were assessed based on their medical records. After applying strict inclusion and exclusion criteria, 125 patients were included in the analysis (Figure 1). All patients received standard sepsis treatment, including fluid resuscitation, vasopressors, hemodynamic management, organ function support, and empirical broad-spectrum antibiotics adjusted based on culture results. Patients requiring mechanical ventilation received sedation and analgesia.

Baseline characteristics, including gender, age, BMI, APACHE II, and SOFA scores, were comparable between groups, as presented in Table 1, with additional details provided in the Supplementary Materials. Immune-related parameters such as CD4^+^ and CD8^+^ counts varied among patients, likely due to differences in infection timing and immune status, as sepsis-induced immunosuppression progresses dynamically.

**TABLE 1:** Comparison of baseline data of patients in Esmolol group and control groups.

	E group (65)	N group (60)	P value
Male	34	35	0.529
Age (y)	76 (49, 86)	74 (34.05, 91.65)	0.840
BMI(kg/m^2^)	23.01 (13.82, 31.85)	23.94 (17.52, 34.98)	0.059
APACHE II	25 (23.76, 26.92)	23 (22.45, 25.19)	0.144
SOFA	7.04 ± 3.07	7.09 ± 2.737	0.929
Underlying diseases			
Diabetes	22 (33.85%)	16 (26.67%)	—
Hypertension	27 (41.54%)	29 (48.33%)	—
Cardiovascular disease	9 (13.85%)	10 (16.67%)	—
Cerebrovascular disease	12 (18.46%)	17 (28.33%)	—
Chronic lung disease	25 (38.46%)	19 (31.67%)	—
Chronic kidney disease	7 (10.77%)	6 (10.00%)	—
Chronic liver disease	9 (13.85%)	7 (11.67%)	—
Connective tissue diseases	16 (24.62%)	12 (20.00%)	—
Tumors	16 (24.62%)	14 (23.33%)	—
No. of underlying diseases	2 (1.88, 2.48)	2 (1.86, 2.43)	0.799
Sources of sepsis			
Lung	61 (93.85%)	55 (91.67%)	—
No. of infected sites	2 (1.57, 2.07)	1 (1.34, 1.78)	0.101
Invasive intubation			
Tracheal intubation	47 (72.31%)	44 (73.33%)	—
CVC	60 (92.31%)	56 (93.33%)	—
Arterial catheterization	9 (13.85%)	6 (10.00%)	—
Urinary catheter	64 (98.46%)	60 (100.00%)	—
Gastrojejunal tube	57 (87.69%)	55 (91.67%)	—
Thoracic/abdominal drains	10 (15.38%)	6 (10.00%)	—
No. of Invasive Catheters	4 (3.53, 4.23)	4 (3.69, 4.20)	0.703
Mechanical ventilation time	9 (8.44, 12.24)	9 (8.02, 10.86)	0.719
ICU-stay (d)	12 (12.23, 16.93)	10 (9.99, 12.45)	0.045
Average hospital-stay (d)	34.439 ± 2.932	12.975 ± 0.913	<0.001
HR_base_	124.68 ± 22.23	112.40 ± 17.84	0.02
Norepinephrine (NE) levels (μg/mL)			
NE (baseline)	437.12 (454.83, 743.81)	354.79 (386.83, 699.92)	0.395
NE (end)	568.63 (513.76, 827.37)	749.44 (867.24, 1528.32)	0.036
ΔNE	54.95 (−0.37, 142.85)	243.13 (372.48,936.34)	<0.001
Total exogenous NE used (mg)	863.77 (162.67, 1564.88)	997.58 (98.16, 2180.16)	0.312

Notes: BMI, body mass index; APACHE II, Acute Physiological Function and Chronic Health Status Score (score); SOFA, Sequential Organ Failure Score (score); ICU, intensive care unit; CVC, central venous catheterization; ΔNE, change in norepinephrine levels from baseline to the final measurement.

The sources of sepsis, including lung (93.85% in the Esmolol group, 91.67% in the control group), abdominal, urinary, blood, skin, soft tissue, and central nervous system infections, were similarly distributed. The use of invasive procedures like tracheal intubation, central venous catheterization, arterial catheterization, urinary catheterization, gastrojejunal tube placement, and thoracic/abdominal drains was comparable between the two groups. The median number of invasive catheters and mechanical ventilation time were also similar. Daily ECGs in the Esmolol group showed no QT interval changes or Esmolol-related side effects.

### 3.2 Main study outcomes

The median ICU stay was significantly longer in the Esmolol group at 12 days (IQR 12.23–16.93) compared to 10 days (IQR 9.99–12.45) in the control group (p = 0.045). Similarly, the average hospital stay was significantly longer for the Esmolol group (34.44 ± 2.93 days) than for the control group (12.98 ± 0.91 days, p < 0.001).

**FIGURE 2:**
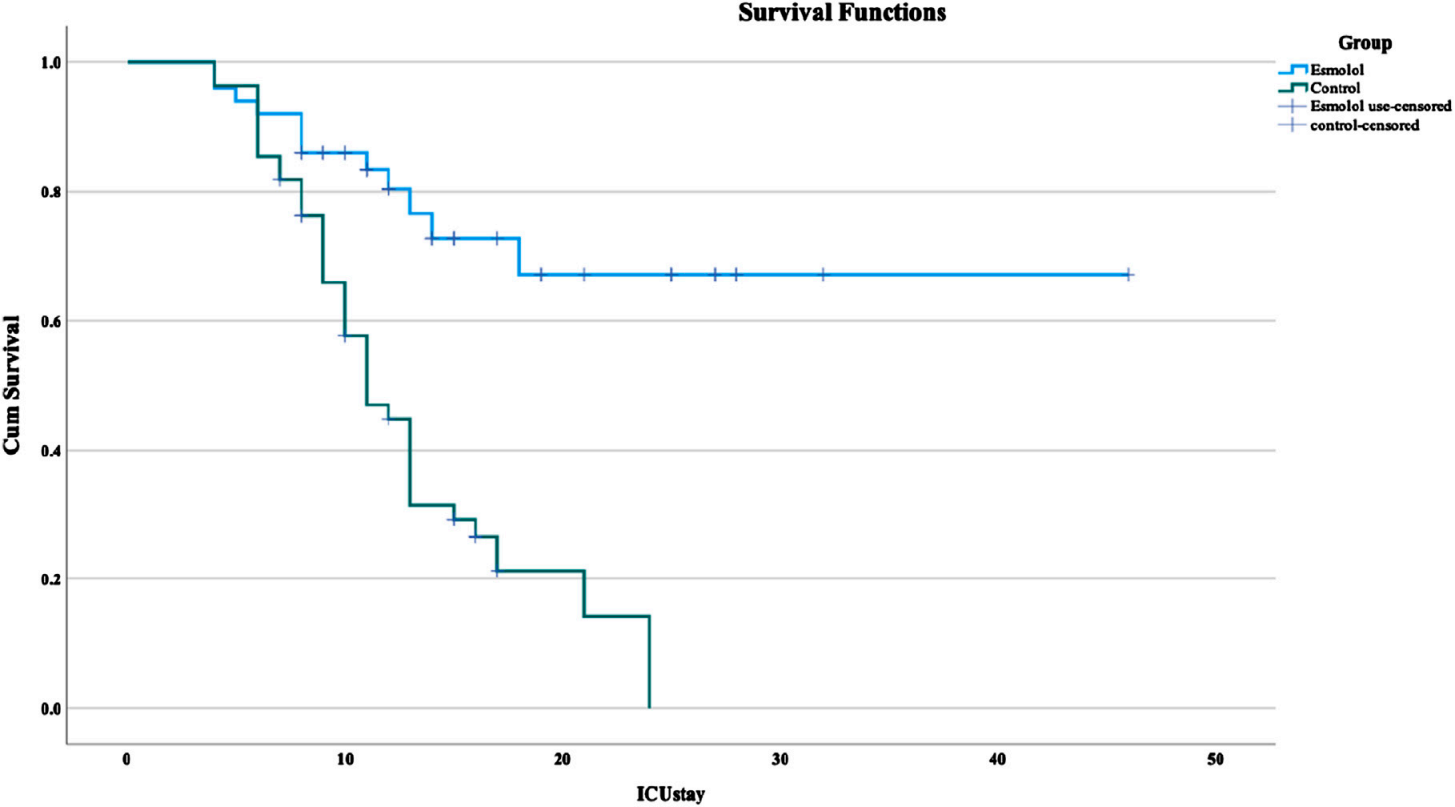
Kaplan-Meier survival analysis of sepsis patients in the ICU. Notes: Cumulative survival is presented using Kaplan-Meier curves, with the x-axis representing ICU stay duration in days and the y-axis representing cumulative survival probability from ICU admission. The esmolol group (blue curve) represents patients treated with esmolol. The control group (green curve) represents patients not treated with esmolol. The horizontal lines at #1 (blue) and #2 (green) indicate post-checkout survival probabilities at specific time points for the respective groups.

Survival rates at 14 days were 80% in the Esmolol group versus 41.67% in the control group (P < 0.01), and at 28 days were 75.38% versus 30.00%, respectively (P < 0.01). Kaplan-Meier survival curves (Figure 2) indicated a significant difference in survival times (chi-square 22.032, P < 0.001).

Baseline heart rates were higher in the Esmolol group (124.68 ± 22.23) compared to the control group (112.40 ± 17.84, p = 0.02). Initial NE levels were similar between groups (Esmolol: 437.12 vs. Control: 354.79, p = 0.395), but by the study’s end, NE levels had significantly increased in the control group (749.44) compared to the Esmolol group (568.63, p = 0.036). The change in NE levels (ΔNE) was also significantly different (Esmolol: 54.95 vs. Control: 243.13, p < 0.001). The total mean exogenous NE used was similar between groups (Esmolol: 863.77 vs. Control: 997.58, p = 0.312).

### 3.3 Multivariate analysis and ROC curve evaluation for predicting sepsis outcomes

Binary logistic regression analysis identified significant predictors of sepsis outcomes, including Esmolol use (B = −3.955, p < 0.001, Exp(B) = 0.019), ICU stay duration (B = 0.211, p = 0.003, Exp(B) = 1.235), and APACHE II scores (B = −0.231, p = 0.018, Exp(B) = 0.794). Other variables like SOFA scores, intubation, initial EC levels, initial platelet counts, CD4^+^, CD8^+^, and infection sites were not significantly associated with outcomes. The model fit was good with −2 log-likelihood = 58.613, Cox & Snell R^2^ = 0.563, and Nagelkerke R^2^ = 0.751. To further assess potential multicollinearity among independent variables, variance inflation factors (VIF) were calculated for all predictors included in the logistic regression model. The VIF values ranged from 1.12 to 2.85, confirming that none of the variables exceeded the conventional cutoff of 10, indicating that multicollinearity was not a concern.

**FIGURE 3:**
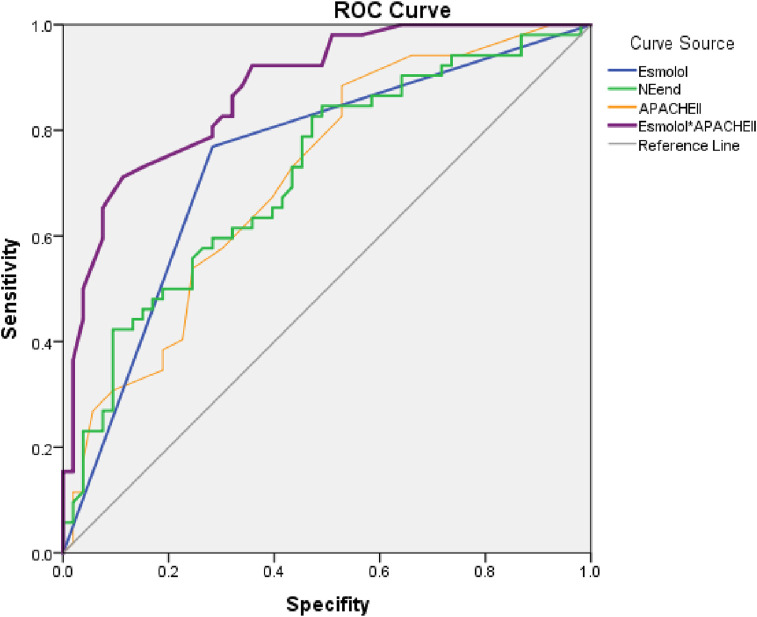
ROC curve analysis for prognostic prediction in sepsis patients. Notes: This figure analyzes the predictive accuracy of prognosis in sepsis patients using different ROC curves. The “Esmolol” curve represents survival predictions based on Esmolol use, coded as 1 for use and 0 for non-use. The “NEend” curve evaluates survival probability using the patient’s final NE levels as a predictor. The “APACHEII” curve predicts prognosis based on the APACHE II score alone. The “Esmolol*APACHEII” curve integrates esmolol use with APACHE II score, demonstrating improved predictive accuracy for sepsis survival compared to individual predictors. The reference line (gray diagonal) represents a random prediction model, where sensitivity equals 1-specificity. The closer a curve is to the upper left corner, the higher the model’s predictive accuracy. The area under the curve (AUC) quantifies predictive performance, with higher AUC values indicating superior prognostic discrimination.

ROC curve analysis was performed to evaluate the predictive accuracy of different models. The AUC for Esmolol use alone was 0.743 (P = 0.049), while NE-end levels and APACHE II scores yielded AUCs of 0.717 (P = 0.05) and 0.712 (P = 0.05), respectively. The combination model integrating Esmolol use, and APACHE II scores demonstrated the highest predictive accuracy, achieving an AUC of 0.878 (P = 0.033, 95% CI: 0.814–0.942). This improvement suggests that integrating Esmolol use with clinical severity scoring significantly enhances survival prediction. The optimal threshold for the combined model was determined to be 25.2, corresponding to a sensitivity of 98.1% and a specificity of 50.9%, further supporting its potential clinical applicability (as shown in Figure 3).

The comparison of different ROC curves further clarified the predictive value of each model. The Esmolol curve assessed survival probability based on Esmolol use as a binary predictor, while the NE-end curve utilized patients’ NE levels at the endpoint to predict survival outcomes. The APACHE II curve estimated prognosis based on APACHE II scores alone. The Esmolol*APACHE II model, which combined Esmolol administration with APACHE II scoring, demonstrated superior predictive power, reinforcing the value of integrating Esmolol treatment with physiological severity assessments. While NE-end levels and APACHE II scores were significant predictors, their predictive ability was lower than that of the Esmolol combination model, highlighting the importance of considering Esmolol use in survival predictions.

The model’s calibration was further validated using the Hosmer-Lemeshow goodness-of-fit test, yielding a chi-square value of 3.366 (P = 0.909). This result indicates that the predicted survival probabilities closely aligned with observed outcomes, confirming that the model was well-calibrated and did not significantly deviate from actual survival distributions. The high P-value supports the robustness of the predictive model and strengthens its applicability in clinical settings. The findings underscore the importance of integrating Esmolol administration with clinical severity scoring in prognostic evaluations and suggest that this approach could enhance the precision of sepsis outcome predictions.

### 3.4 Esmolol usage during E group

In the Esmolol group, the total dose of Esmolol was 9 g (range 7.31–17.18) in the death group and 16 g (range 14.08–23.29) in the survival group, though this difference was not statistically significant (P = 0.211). The duration of Esmolol use was significantly longer in the survival group (6 days, range 5.8–7.88) compared to the death group (4 days, range 3.22–5.95) (P = 0.019). The average daily rate of Esmolol was similar between groups (110.25 mg/h in the death group and 94.33 mg/h in the survival group, P = 0.266).

Heart rate after Esmolol administration was significantly lower in the survival group (95.25 bpm, range 94.79–103.63) compared to the death group (119.50 bpm, range 106.78–132.14) (P = 0.002). Furthermore, a higher proportion of patients in the death group (12.50%) received delayed Esmolol treatment (administered more than 5 days after ICU admission), compared to only 4.1% in the survival group (P = 0.043).

### 3.5 Mechanistic insights into Esmolol’s clinical effects

#### 3.5.1 Biochemical results comparison between esmolol and control groups

Baseline characteristics, including complete blood count parameters, inflammatory markers (e.g., CRP, PCT, ESR, ferritin, interleukins, TNF, IFN), cardiac markers, liver function tests, renal function markers, and amylase, lipase showed no significant differences between the E and N groups immediately after ICU admission.

However, significant differences were observed after Esmolol treatment. Esmolol reduced heart rate, which lowered myocardial and muscle tissue oxygen consumption, as indicated by decreased MYO levels (ΔMYO: 9.25 vs. 320.10, p = 0.023) and Lac levels (ΔLac: −0.8 vs. −0.05, p = 0.005). Additionally, a decrease in NE levels (ΔNE) resulted in reduced NE-end levels (p = 0.036), indicating reduced sympathetic nervous system activity. Renal function improved, evidenced by lower Cr levels (ΔCr: −3.2 vs. 30.55, p = 0.006). The Esmolol group also showed a greater reduction in CRP levels (ΔCRP: −55.135 vs. −5.52, p = 0.002) and a less pronounced decrease in Alb levels (ΔAlb: −1.70 vs. −4.45, p = 0.027). These changes are detailed in Supplementary Table S1.

#### 3.5.2 Dynamic changes in immune-related indicators during Esmolol treatment

During Esmolol treatment, significant changes in immune-related indicators were observed. A GEE analysis, adjusting for time variations, APACHE II score, and age, revealed a significant reduction in median PCT levels: 1.25 (95% CI 5.74, 19.54) for the Esmolol group versus 3.78 (95% CI 10.80, 17.85) for the control group (P = 0.005).

Interleukins also showed notable changes. Each additional unit of Esmolol resulted in an average decrease of 22.254 units in IL-6 (P = 0.009), and significant reductions in IL-10 levels through its effect on NE levels (P = 0.002). TNFα levels decreased with Esmolol use, with each 1-unit increase in NE following Esmolol administration reducing TNFα by 31.587 units. An inverse relationship was observed between NE and IFN-γ levels (Mean 6.43 vs. 12.33, P = 0.008), suggesting that Esmolol-mediated reduction in NE levels may suppress IFN-γ production, possibly through β1AR blockade, which typically enhances Th1-mediated immune responses. This contrasts with TNFα, which is a pro-inflammatory cytokine often elevated in sepsis, and its reduction alongside NE may indicate a balancing effect between pro-inflammatory and immune-regulatory pathways during Esmolol treatment.

Additionally, Esmolol significantly impacted the temporal dynamics of the CD64 granulocyte index, reducing it by 14.404 units per Esmolol unit increase, an effect that remained significant after adjustments for APACHE II scores and age (P = 0.007). However, Esmolol did not have a direct significant impact on the CD64 monocyte index dynamics (P = 0.226).

#### 3.5.3 Effect on CD4^+^ and CD8^+^ T-cell counts

Neither Esmolol use nor NE levels independently showed a significant effect on CD4^+^ and CD8^+^ T-cell counts. However, significant correlations were found when considering baseline CD4^+^ levels before Esmolol use. Patients with baseline CD4^+^ levels ≤200 cells/µL showed further decreases in CD4^+^ counts with Esmolol treatment, whereas those with baseline CD4^+^ levels >200 cells/µL exhibited significant increases.

Specifically, in patients with baseline CD4^+^ levels ≤200, Esmolol was associated with a further significant decrease in CD4^+^ T-cell count, with counts dropping to 122.49 (NE↑) and 98.06 (NE↓) (P < 0.01). Conversely, in patients with baseline CD4^+^ levels >200, CD4^+^ T-cell counts increased significantly with Esmolol treatment, rising to 289.8 (NE↑) and 346.06 (NE↓) (P < 0.01). The mean baseline CD4^+^ count of 251.61 in the surviving patients on Esmolol was higher than the mean of 106.92 in the deceased group, but the difference was not significant (P = 0.08) (shown in Table 2).

**TABLE 2:** Comparative analysis of esmolol usage and outcomes between death and survival groups.

Category	Death group (16)	Survival group (49)	P value
Dosage of E total (g)	9 (7.31,17.18)	16 (14.08, 23.29)	0.211
Duration of E (d)	4 (3.22, 5.95)	6 (5.8, 7.88)	0.019
Average daily rate of E (mg/h)	110.25 (90.04, 131.72)	94.33 (88.68, 117.84)	0.266
HR before E	132.5 (126.45, 140.71)	125.5 (124.81, 130.11)	0.122
HR after E	119.50 (106.78, 132.14)	95.25 (94.79, 103.63)	0.002
E delayed administration (>5 days)	2 (12.50%)	2 (4.1%)	0.043
CD4^+^ (initial)	95.0 (68.63, 145.20)	148.5 (169.52, 333.69)	0.080

Notes: Dosage of E total (g): Total amount of Esmolol administered during the treatment period.

Duration of E (d), Total number of days Esmolol was administered; Average daily rate of E (mg/h), Average hourly rate of Esmolol administration; HR before E, Heart rate measured before Esmolol administration; HR after E, Heart rate measured after Esmolol administration; E delayed administration (>5 days), Number of patients receiving Esmolol more than 5 days post-sepsis onset.

**FIGURE 4:**
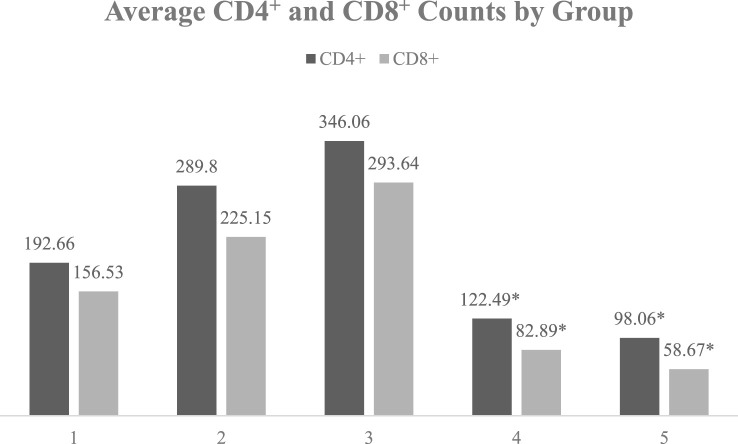
Average CD4^+^ and CD8^+^ counts by group in sepsis patients. Notes: The graph shows the average CD4^+^ and CD8^+^ counts for each group, emphasizing the trends and changes in these immune-related indicators among the different groups. Group 1: Control group (N group); Group 2: Esmolol group with baseline CD4^+^ > 200 and increased NE levels; Group 3: Esmolol group with baseline CD4^+^ > 200 and decreased NE levels; Group 4: Esmolol group with baseline CD4^+^ ≤ 200 and increased NE levels; Group 5: Esmolol group with baseline CD4^+^ ≤ 200 and decreased NE levels * Indicates a significant difference compared to group N (p < 0.01). Groups with baseline CD4 > 200 were highlighted in green, as increases in CD4^+^ and CD8^+^ counts were observed with Esmolol treatment, indicating beneficial effects. Conversely, groups with baseline CD4^+^ ≤ 200 were highlighted in yellow, where decreases in CD4^+^ and CD8^+^ counts were observed with Esmolol treatment, indicating significant differences compared to the control group (N).

For CD8^+^ T-cell counts, a significant difference was observed between the control group (156.53) and the group with decreased NE levels following Esmolol administration (NE↓CD4^+^>200: 293.64; P = 0.004). In patients with baseline CD4^+^ levels ≤200, Esmolol treatment was associated with significantly lower CD8^+^ T-cell counts compared to the control group, with counts at 82.89 (NE↑) and 58.67 (NE↓) (P = 0.006 and P = 0.014, respectively). However, in patients with baseline CD4^+^ levels >200, Esmolol treatment led to higher CD8^+^ T-cell counts compared to the control group, with counts at 225.15 (NE↑) and 293.64 (NE↓) (P = 0.042 and P < 0.001, respectively) (As shown in Figure 4).

## 4 Discussion

Sepsis is associated with sympathetic nervous system overactivation, increased cardiac workload, and inadequate tissue perfusion. Esmolol improves cardiovascular function by reducing NE levels and lactate. Previous studies indicated that Esmolol shortens mechanical ventilation duration and reduces 28-day mortality (Shang et al., 2016; Zhang et al., 2022). Our findings align with these, showing longer survival and better outcomes, linked to APACHE II scores, improved organ function, and reduced inflammation. The significant improvement in clinical outcomes with Esmolol in our study can be attributed to several key factors: early administration, higher baseline CD4^+^ levels in Esmolol-treated patients, and prolonged use of Esmolol. These factors, although not prospectively optimized, were identified as significant contributors to the observed benefits in our retrospective analysis.

### 4.1 Esmolol’s role as a β1 adrenergic receptor blocker with immunomodulatory properties

Esmolol, by blocking β1 receptors, exerts significant immunomodulatory effects by both directly regulating immune cell activity and indirectly reducing excessive sympathetic activation and NE levels. This dual mechanism contributes to the rebalancing of the immune system in sepsis, potentially mitigating both excessive inflammation and immune exhaustion.

Experimental data indicate that Esmolol reduces T cell apoptosis and restores Th1/Th2 balance, an effect linked to the Akt/Bcl-2/Caspase-3 pathway, with additional inhibition of p-Erk1/2 signaling, thereby preserving Th1-driven bacterial clearance (Ma et al., 2023). Beyond its role in T cell regulation, Esmolol has been associated with modulation of NF-κB activity, which contributes to reduced IL-6 and TNF-α levels, promoting a more controlled inflammatory response (Guo et al., 2020).

In addition to these immune-modulating effects, Esmolol has demonstrated protective benefits in bacterial infections. Studies on multidrug-resistant *Pseudomonas aeruginosa* have shown improved survival when Esmolol is combined with antibiotic therapy (Dimopoulos et al., 2015). The clinical relevance of these effects is further supported by findings demonstrating that Esmolol treatment significantly reduces 28-day mortality and ICU stay duration in septic patients (Wei et al., 2024).

These observations suggest that Esmolol’s therapeutic potential depends on both disease progression and individual immune status, requiring further optimization in sepsis management strategies.

### 4.2 Effects of Esmolol on T cells

The immunomodulatory properties of Esmolol are closely linked to its role in T cell regulation, particularly through β1AR blockade, which mitigates NE-induced T cell apoptosis and Th2 polarization.

#### 4.2.1 Esmolol reduces T lymphocyte apoptosis and restores Th1/Th2 balance

Excessive β1-adrenergic activation in sepsis contributes to T cell apoptosis and immune suppression. Experimental findings indicate that Esmolol downregulates Caspase-3 activation, upregulates Bcl-2, and inhibits Erk1/2, collectively leading to improved T cell survival and a rebalancing of Th1/Th2 differentiation, which is essential for pathogen clearance (Ma et al., 2023).

Beyond β1AR blockade, Esmolol has been shown to influence the α7 nicotinic acetylcholine receptor (α7 nAChR) pathway, a mechanism critical for immune regulation. Increased choline acetyltransferase (ChAT) expression, enhanced STAT3 activation, and suppression of NF-κB signaling following Esmolol treatment are associated with reductions in IL-1, IL-6, and TNF-α production, ultimately improving immune cell function and reducing apoptosis in septic conditions (Takayanagi et al., 2012).

#### 4.2.2 NE modulates IFN-γ and TNF-α production through β-adrenergic receptors

NE exerts a well-characterized inhibitory effect on IFN-γ production via β1AR activation, a suppression that can be reversed with β1AR antagonists (Takenaka et al., 2016). Additionally, β2AR activation by NE leads to reduced IL-12 secretion from dendritic cells, further dampening Th1 differentiation and IFN-γ production (Wang et al., 2025).

In contrast, TNF-α regulation is less directly controlled by NE, as its secretion is primarily governed by NF-κB-mediated inflammatory signaling. Although NE can inhibit NF-κB via β2AR, the overall regulation of TNF-α remains complex, with multiple inflammatory cytokines such as IL-1 and IL-6 playing key roles (Takenaka et al., 2016). Esmolol’s effects on TNF-α and IL-6 appear to be independent of NE suppression, instead acting through α7 nAChR/STAT3-mediated NF-κB inhibition, reinforcing its broader immunoregulatory potential (Takayanagi et al., 2012).

#### 4.2.3 Why is the negative correlation between NE and IFN-γ more pronounced than TNF-α?

The stronger inverse relationship between NE and IFN-γ, compared to NE and TNF-α, is explained by differences in their regulatory pathways. IFN-γ suppression by NE is a direct effect mediated by β1AR on Th1 cells, whereas TNF-α is modulated by a broader inflammatory network, making its dependence on adrenergic signaling less pronounced (Takayanagi et al., 2012). The additional suppression of IL-12 via β2AR further reinforces NE’s inhibitory effect on IFN-γ production, whereas TNF-α secretion remains less directly affected by NE levels (Takenaka et al., 2016). These findings suggest that Esmolol’s effects on immune function are more pronounced in pathways directly linked to β1AR signaling, such as Th1 responses, whereas TNF-α regulation is influenced by broader inflammatory mechanisms.

#### 4.2.4 Esmolol’s effects depend on baseline immune status

The response to Esmolol treatment appears to be dependent on the patient’s initial immune status. Experimental models indicate that β1AR activation suppresses CD4^+^ T cell function in sepsis, leading to immune exhaustion (Durand et al., 2022). In clinical observations, patients with higher baseline CD4^+^ counts exhibited improved immune recovery following Esmolol treatment, while those with severe lymphopenia showed no clear benefit, suggesting that the efficacy of β1AR blockade may vary based on pre-existing immune function. These findings highlight the need for immune profiling in Esmolol-treated patients, particularly through biomarkers such as CD4^+^/CD8^+^ ratios and cytokine signatures, to identify those most likely to benefit from its immunoregulatory effects.

### 4.3 Optimal timing of esmolol use in sepsis management

The timing of Esmolol administration plays a crucial role in determining its therapeutic impact. During early sepsis, excessive SNS activation and high NE levels initially contribute to immune dysregulation and inflammation, but prolonged exposure promotes T cell apoptosis and bacterial proliferation, exacerbating immune dysfunction.

#### 4.3.1 Early use of esmolol improves survival in sepsis

Early β1AR blockade has been shown to stabilize cardiac function, restore vascular responsiveness, and reduce NF-κB activation, leading to improved survival in experimental septic shock (Kimmoun et al., 2015). Clinical meta-analyses confirm that Esmolol reduces 28-day mortality and ICU stay duration by controlling excessive inflammation and hemodynamic instability (Wei et al., 2024). Our findings align with these observations, demonstrating improved survival and organ function in patients receiving early Esmolol treatment.

#### 4.3.2 Esmolol in late-stage sepsis: addressing immune paralysis

As sepsis progresses, immune dysfunction shifts towards immune paralysis, driven by MDSC activation and IL-10 overproduction. While β2AR activation enhances IL-10 secretion, limiting early inflammation, prolonged IL-10 elevation contributes to immune exhaustion and increased infection risk (Agac et al., 2018).

Our study found no evidence that Esmolol worsens immune suppression. Instead, treated patients maintained better immune function, suggesting that β1AR blockade helps modulate immune suppression without excessive dampening of protective responses. Further investigation is needed to determine how Esmolol influences IL-10 pathways and immune homeostasis.

Given this variability, a biomarker-driven approach to Esmolol therapy is necessary. Monitoring CD4^+^ counts, IL-10 levels, and MDSC activity may help optimize timing and dosing, ensuring maximal benefit while avoiding immunosuppressive risks.

### 4.4 Limitations of the study

This study is limited by its retrospective design and single-center data, which may affect the generalizability of the results. Our center’s specific demographic and clinical practices might not represent wider healthcare settings. Patients in this study were severely ill, indicated by an average APACHE II score of 24 and a high mortality rate, typical for our ICU. Despite using multivariate analysis to control for confounding variables, not all potential confounders such as baseline health status, comorbidities, and concurrent medications could be fully accounted for, which may significantly influence the outcomes and the observed effects of Esmolol. The retrospective nature of the study also means that biases in data recording and treatment administration might have been introduced.

The study did not fully explore the complexities of Esmolol’s effects on the immune system across different stages of sepsis, highlighting the need for more detailed studies to understand its mechanisms. Furthermore, due to the COVID-19 pandemic, there were significant delays in data collection and clinical activities. Although cases of viral sepsis, including COVID-19, were excluded to maintain the homogeneity of the study population, the pandemic led to delays that impacted the overall data collection process and may have introduced additional variability.

Future research should include multicenter prospective studies with larger and more diverse populations to confirm these findings and improve applicability across different settings. These studies should also explore Esmolol’s mechanistic pathways, optimal dosing, and administration strategies. Employing a double-blind design, if feasible, could help validate the findings from this retrospective study and reduce potential biases.

## 5 Conclusion

This study highlights Esmolol’s significant role in sepsis management through immune modulation and inflammation reduction. Esmolol stabilizes cardiovascular function and positively influences T-cell dynamics, crucial for effective sepsis management. Proper timing and dosage are essential to maximize benefits and minimize risks. Future multicenter prospective trials should confirm these findings and further explore Esmolol’s mechanisms. The significant survival and immunomodulatory benefits suggest that β1-adrenergic blockade could enhance sepsis treatment protocols, especially for patients with strong baseline immune function.

## Data Availability

The original contributions presented in the study are included in the article/Supplementary Material, further inquiries can be directed to the corresponding author.

## Glossary

APACHE II: Acute Physiology and Chronic Health Evaluation II
SOFA: Sequential Organ Failure Assessment
ICU: Intensive Care Unit
CRRT: Continuous Renal Replacement Therapy
ECMO: Extracorporeal Membrane Oxygenation
NE: Norepinephrine
ΔNE: Change in Norepinephrine
CD4: Cluster of Differentiation 4
CD8: Cluster of Differentiation 8
IL-6: Interleukin 6
IL-10: Interleukin 10
TNF-α: Tumor Necrosis Factor Alpha
IFN-γ: Interferon Gamma
PCT: Procalcitonin
Lac: Lactic Acid
WBC: White Blood Cells
LC: Lymphocyte Count
EC: Eosinophil Count
HCT: Hematocrit
CRP: C-Reactive Protein
MYO: Myoglobin
cTnI: Cardiac Troponin I
D-D: D-Dimer
ESR: Erythrocyte Sedimentation Rate
nCD64: Neutrophil CD64
mCD64: Monocyte CD64
AST: Aspartate Aminotransferase
ALT: Alanine Aminotransferase
LDH: Lactate Dehydrogenase
TBIL: Total Bilirubin
MAP: Mean Arterial Pressure
BMI: Body Mass Index
HR: Heart Rate
EF: Ejection Fraction
SNS: Sympathetic Nervous System
Th1: T helper 1 cells
Treg: Regulatory T cells
MDSCs: Myeloid-derived Suppressor Cells
ROC: Receiver Operating Characteristic
AUC: Area Under the Curve
NYHA: New York Heart Association
SIRS: Systemic Inflammatory Response Syndrome
EHR: Electronic Health Record
GEE: Generalized Estimating Equations
