# Supplementary material for: Esmolol improves sepsis outcomes through cardiovascular and immune modulation
Source: Front Pharmacol. 2025 May 12;16:1498227. doi: 10.3389/fphar.2025.1498227 (PMC12104586; doi:10.3389/fphar.2025.1498227)
Supplement: Supplementary file 1 [file Table1.docx]

**Supplementary materials**

**Table: Comparison of biomarkers of patients in Esmolol group and control groups**

|  | **E group（65）** | **N group (60)** | **P value** |
| --- | --- | --- | --- |
| Lac (mmol/L) | 2.30 (2.22, 3.94) | 1.95 (1.78, 2.67) | 0.018 |
| WBC (×10^9^) | 9.36 (8.64, 15.07) | 10.72 (9.64, 13.21) | 0.835 |
| LC (×10^9^) | 0.59 (-18.16, 55.78) | 0.45 (0.39, 0.62) | 0.715 |
| EC (×10^9^) | 0.012 (0.02, 0.08) | 0.003(0.005, 0.039) | 0.394 |
| HCT (L/L) | 33.04±8.34 | 32.96±8.69 | 0.532 |
| PLT (×10^9^) | 158.50 (151.73, 251.27) | 152.50 (133.99, 182.36) | 0.354 |
| CRP (mg/L) | 91.36 (80.44, 146.42) | 76.08 (62.53, 120.09) | 0.112 |
| PCT (ng/ml) | 1.01 (-0.16, 23.63) | 1.86 (2.53, 12.34) | 0.125 |
| MYO (ng/ml) | 159.9 (128.10, 1063.15) | 179.7 (180.61, 750.85) | 0.922 |
| cTnI (ug/L) | 31.3 (44.38, 353.05) | 47.95 (138.74, 629.59) | 0.284 |
| D-Dimmer (ng/ml) | 3.71 (3.36, 7.91) | 6.47 (6.87, 16.19) | 0.206 |
| ESR (mm/h) | 55.5 (43.04, 69.49) | 54.5 (35.38, 61.03) | 0.198 |
| Ferritin (ng/ml) | 770.55 (528.92, 2954.23) | 1054.25(820.32, 2841.57) | 0.769 |
| CD4^+^ (cells/ul) | 131 (123.78, 314.49) | 107.50 (102.58, 158.95) | 0.768 |
| CD8^+^(cells/ul) | 71.5 (99.67, 224.46) | 82 (58.25, 159.57) | 0.847 |
| nCD64 (MFI) | 36.48 (30.42, 62.21) | 19.53 (24.28, 61.08) | 0.343 |
| mCD64 (MFI) | 175.04 (147.57, 212.73) | 161.83 (145.22, 219.96) | 0.917 |
| IL6 (pg/ml) | 88.72 (166.80, 859.85) | 108.74 (-2725.45, 8730.59) | 0.287 |
| IL10 (pg/ml) | 4.11 (-4.74, 79.33) | 6.55 (13.29, 44.18) | 0.893 |
| TNF (pg/ml) | 1.79 (1.19, 3.96) | 1.68 (1.44, 2.17) | 0.351 |
| IFN (pg/ml) | 2.86 (-1.79, 23.88) | 2.97 (-1.11, 31.30) | 0.742 |
| Urea (mmol/L) | 10.08 (8.57, 14.30) | 9.17 (8.06, 11.65) | 0.707 |
| Cr (μmol/L) | 78.6 (69.85, 138.96) | 66.2 (70.87, 115.71) | 0.684 |
| Alb (U/L) | 29.33±5.10 | 30.19±4.06 | 0.681 |
| AST (U/L) | 48.5 (49.55, 129.58) | 58 (42.7, 271.12) | 1.000 |
| ALT (U/L) | 21.5 (20.6, 86.66) | 22 (18.92, 182.37) | 0.807 |
| LDH (U/L) | 285.5 (290.06, 414.94) | 359.5 (372.46, 667.83) | 0.959 |
| TBIL (U/L) | 15.35 (13.51, 41.65) | 16.8 (15.64, 32.18) | 0.990 |
| Amylase (U/L) | 59.5 (64.47, 139.26) | 77 (68.80, 175.79) | 0.236 |
| Lipase (U/L) | 28.75 (26.57, 69.95) | 31.3 (30.75, 84.106) | 0.377 |
| ΔLac (mmol/L) | -0.8 (-1.93， -0.19） | -0.05 (0.1, 2.07) | 0.005 |
| ΔCRP (mg/L) | -55.135 (-82.85, -38.73) | -5.52 (-30.72, 26.97) | 0.002 |
| ΔAlb (U/L) | -1.70 (-3.32, -0.10) | -4.45 (-6.60, -2.97) | 0.027 |
| ΔCr (μmol/L) | -3.2 (-27.88, 42.13) | 30.55 (17.78, 94.47) | 0.006 |
| ΔMYO (ng/ml) | 9.25 (-1018.63, 1610.67) | 320.10 (260.95, 8302.07) | 0.023 |

***Notes:*** *Lac: Lactic Acid; WBC: White Blood Cells; LC: Lymphocyte Count; EC: Eosinophil Count; HCT: Hematocrit; PLT: Platelets; CRP: C-Reactive Protein; PCT: Procalcitonin; MYO: Myoglobin; cTnI: Cardiac Troponin I; D-D: D-Dimer; ESR: Erythrocyte Sedimentation Rate; CD4: Cluster of Differentiation 4; CD8: Cluster of Differentiation 8; nCD64: Neutrophil CD64; mCD64: Monocyte CD64; IL6: Interleukin 6; IL10: Interleukin 10; TNF: Tumor Necrosis Factor; IFN: Interferon; Cr: Creatinine; Alb: Albumin; AST: Aspartate Aminotransferase; ALT: Alanine Aminotransferase; LDH: Lactate Dehydrogenase; TBIL: Total Bilirubin; Δ: Difference between last and first results.*
